# Safety and Efficacy of Stereotactic Ablative Radiotherapy for Ultra-Central Lung Cancer

**DOI:** 10.3389/fonc.2022.868844

**Published:** 2022-04-29

**Authors:** Bin Wang, Yang Dong, Xuyao Yu, Fengtong Li, Jingsheng Wang, Huaming Chen, Zeqian Niu, Yongchun Song, Zhiyong Yuan, Zhen Tao

**Affiliations:** ^1^ Department of Radiation Oncology, Xijing Hospital, Fourth Military Medical University, Xi’an, China; ^2^ Department of Radiation Oncology, CyberKnife Center and Tianjin’s Clinical Research Center for Cancer, Key Laboratory of Cancer Prevention and Therapy, National Clinical Research Center for Cancer, Tianjin Medical University Cancer Institute and Hospital, Tianjin, China

**Keywords:** stereotactic body radiotherapy, cyberknife, ultra-central lung cancer, outcomes, toxicity

## Abstract

**Background:**

Ultra-central lung cancer (UCLC) is difficult to achieve surgical treatment. Over the past few years, stereotactic ablative radiotherapy (SABR) or stereotactic body radiotherapy (SBRT) obviously improved the clinical efficacy and survival of UCLC patients. However, the adapted scheme of radiation therapy is still controversial. For this, a single arm retrospective analysis was performed on UCLC patients treated with SBRT.

**Material and Methods:**

We retrospectively studied primary UCLC patients who were treated with SBRT of 56 Gy/6-8f between 2010 and 2018. UCLC was defined as planning target volume (PTV) touching or overlapping the proximal bronchial tree, trachea, esophagus, heart, pulmonary vein, or pulmonary artery within 2 cm around the bronchial tree in all directions.

**Results:**

A total of 58 patients whose median age was 68 years (range, 46-85) were included in our study, 79.3% of whom did not undergo any previous therapy. The median dose of the PTV was 77.8 Gy (range, 43.3-91.8), and the median PTV of tumors was 6.2 cm^3^ (range, 12.9-265.0). With a median follow-up of 57 months (range, 6-90 months), the median cumulative overall survival (OS) rate was 58 months (range, 2-105). In addition, the 1-year, 2-year and 5-year OS rates were 94.7%, 75.0% and 45.0%, respectively. In our univariable analysis (p=0.020) and multivariate analysis (p=0.004), the OS rate was associated with the PTV. The 5-year OS rates for PTV <53.0 cm^3^ and PTV ≥53.0 cm^3^ were 61.6% and 37.4%, respectively. Regarding toxicity after SBRT, there were two cases (3.5%) with grade ≥3 adverse events, of which 1 case died of sudden severe unexplained hemoptysis.

**Conclusions:**

Patients with UCLC can benefit from SBRT at a dose of 56 Gy/6-8f. On the other hand, smaller PTV was associated with superior outcomes, and the cure difference needs to be validated by prospective comparative trials.

## Introduction

For several years, surgery has been a preferred alternative for early-stage lung cancer; however, in some cases, such as elderly patients or patients with poor physical conditions and tumors closing or overlapping the mediastinal structure, surgery is not feasible. For these patients, radiotherapy could be a suitable choice because it is non-invasive and has fewer side-effects. SABR or SBRT, as an accurate and effective treatment option, is commonly applied in non-small cell lung cancer (NSCLC) (1–5). However, the safety, and efficacy of SBRT in patients with UCLC are currently controversial.

From an early Indian study, the Radiation Therapy Oncology Group (RTOG) 0236 trial defined centrality as a tumor either touching or located within the 2 cm zone of the proximal bronchial tree in all directions. Although the dose of 60 Gy divided into 3 fractions (f) was tolerable in all patient groups, the treatment plan was not suitable for patients with central lung tumors (6–9). Another recent clinical trial named “RTOG 0813” designed different doses in 5 fractions, and the results showed that the two-year overall survival (OS) rates of 60 Gy/5 f (33 cases) and 57.5 Gy/5 f (38 cases) were 72.7% and 7.2%, respectively. Simultaneously, there was relatively high radiotherapy-related toxicity in the trial (7). The retrospective analysis by Cong Yang et al. showed that SBRT with a dose of 35 Gy/4–6f for patients with advanced-stage ultra-central NSCLC was effective and tolerable (10).

Cyberknife (CK) a real-time target tracking technology, allowed SBRT to precisely deliver high-energy rays from omnidirectional angles to realize a more efficient and shorter treatment scheme (11).UCLC, as a subgroup with the highest risk of central lung cancer, lacks a standard dose prescription. Although the UCLC case was involved in the RTOG 0813 trial, the results of a few cases were not of great significance. To identify effective radiotherapy regimens, we retrospectively investigated the clinical application results of UCLC patients over ten years in our institution.

Based on our experience and dates, we hypothesize that UCLC patients could benefit from SBRT at a dose of 56/6-8f Gy. The results of this series may help to better understand the management of UCLC.

## Material and Methods

### Patient Selection

The present study was approved by our hospital ethics board and obtained for experimentation with human subjects (Batch number: EK2020204). All patients in this study had signed informed consent. The study was carried out in conformity with the principles of the Helsinki Declaration, Good Clinical Practice, and the study protocol. The privacy rights of human subjects always were observed between 2010 and 2018, there were 64 patients with stage T1N0 to T3N0 UCLC. All these patients were treated with CK at 56 Gy at our hospital. Tumor staging was performed referring to the 7th edition of the American Joint Committee on Cancer (AJCC) staging system assessed by Computed Tomography (CT) scans of the chest and abdomen or positron emission tomography (PET) (12). Patients with a previous history of pneumonia or adult respiratory distress syndrome were not included. Three patients with multiple lesions and two patients with missing information were also excluded. Finally, there were 58 patients with single ultra-central lesions in our cohort. The collection of data was performed based on a retrospective chart review. Baseline patient characteristics are presented in **Table 1**.

**Table 1:** Characteristics of the 58 patients and tumors.

Characteristics	Frequency (%) N = 58
Age, years	
Mean ± SD	67 ± 9.87
Median (range)	68 (46,85)
Sex	
Male	43 (74.1%)
Female	15 (25.9%)
Smoking status	
Never	12 (22.0%)
Past or current	46 (78.0%)
KPS	
Median (range)	80 (70-100)
ECOG	
0-1	53 (91.4%)
≥2	5 (8.6%)
Pre-SBRT symptoms	
None	12 (20.7%)
Cough	43 (74.1%)
Hemoptysis	18 (31.0%)
Chest tightness	18 (31.0%)
Histology	
Squamous carcinoma	16 (27.6%)
Adenocarcinoma	11 (19.0%)
Others	7 (12.0%)
No pathology available	24 (41.4%)
T stage	
T1	20 (34.5%)
T2	33 (56.9%)
T3	5 (8.6%)
PTV (cm³)	
Median (range)	60.2 (12.9-265.0)
<53.0	21 (36.2%)
≥53.0	37 (63.8%)
Previous treatment	
None	46 (79.3%)
Chemotherapy	8 (13.8%)
Others	4 (6.9%)
Treatment scheme	
Radiotherapy alone	50 (86.2%)
Concurrent target therapy	1 (1.7%)
Consolidation radiotherapy	7 (12.1%)

PTV, planning target volume.

### Treatment

SBRT was delivered using the CK precisely. All patients received 56 Gy in different fractions. The ordinary fractions were 8 Gy *7 (n=31) or 7 Gy *8 (n=25), and two patients received 56 Gy/6f. In terms of previous treatment, 46 patients (79.3%) did not receive any tumor-related therapy before, 8 (13.8%) patients underwent chemotherapy, 3 patients (5.2%) underwent excision of non-radial tumors (two of which were independent tumor resections and the other was a resection of carcinoma in situ) and one patient (1.7%) received immunotherapy.

Treatment was performed with robotic SBRT using CK at our cancer institute and hospital. Quality assurance of dose and beam accuracy was performed daily. All patients were immobilized with customized dual vacuum immobilization devices and underwent 4-dimensional (4D) noncontrast planning CT. In SBRT, GTV was defined as the visible extent of the tumor. For patients receiving consolidation radiotherapy, GTV was considered the extent of the tumor before chemotherapy. For ITV, except for the 4D-CT respiratory phase delineation, the location of the lung lobe where the tumor is located was also considered. We then expanded 5 mm in all directions to account for the uncertainty of the setting, which formed the PTV. Fractionated RT schedules were based on the tumor volume, age, and physical condition of the patients and were determined by the attending physician after assessing the risk of treatment. BED _10_ = nd [1 + d/(α/β)], in units of Gy. EQD_2_= D× (d + α/β)/(2.0 + α/β) (n and d represent the number of fractions and the fraction size, respectively, and α/β is assumed to be 10 Gy for tumors). The Dmax, the maximum dose of a structure at a point, was calculated by the planning system.

### Statistical Analysis

We defined OS as the interval from the date of treatment to the date of death or the last follow-up visit. Progression-free survival (PFS) was measured from the date of treatment to the date of disease progression, relapse, death, or the last follow-up visit. The tumor local control (LC) rate was calculated from the first treatment date until local tumor progression/metastasis, the last time of follow-up or death. Local responses to treatment were classified according to the modifications of the Response Evaluation Criteria in Solid Tumors (RECIST) (13). Acute toxicities were defined as treatment-related side effects that occurred within 90 days after the first fraction, whereas late toxicities occurred after this time. All toxicities were evaluated in a multidisciplinary environment, and the determination of related toxicities was based on the Common Terminology Criteria for Adverse Events version 5.0 (CTCAE 5.0).

OS, PFS, and LC curves were estimated by using the Kaplan‐Meier method and compared by the log‐rank test. Multivariate analyses were performed using the Cox proportional hazards model. Two-sided P-values of 0.05 was considered statistically significant. All the above statistical analyses were performed with SPSS software (SPSS Standard version 19.0, SPSS Inc., Chicago, IL, USA) and MedCalc statistical software (MedCalc-version 20.027).

## Results

### Patients and Tumors

A total of 58 patients (43 males and 15 females), with a median age of 68 years (range, 43-85), were included in this study. Before CK therapy, 12 (20.7%) patients did not have any symptoms, and 43 (74.1%) patients had a mild cough. Eighteen (31.0%) patients had bloody sputum, and 18 (31.0%) patients had chest tightness. During the treatment, 50 (86.2%) patients received CK alone, and 1 (1.7%) patient received targeted agent treatment. To improve prognosis, the other 7 (12.1%) patients received consolidation radiotherapy.

Regarding the aspects of tumor characteristics, the median PTV was 60.2 (12.9-265) cm^3^. Regarding the pathological classification, 16 (27.6%) cases were squamous carcinoma, 11 (19.0%) cases were adenocarcinoma, 7 (12.0%) were other pathological types, and 24 (41.4%) cases were unavailable. Furthermore, the numbers of T1, T2, and T3-stage tumors were 20 (34.5%), 33 (56.9%), and 5 (8.6%), respectively. The characteristics of the patients and tumors are shown in **Table 1**.

### Treatment

The prescribed dose at 56 Gy was delivered in 6 (3.5%), 7 (53.4%) and 8 (43.1%) fractions with median BED_10 =_ 100.8 (95.2-111.5) and median EQD_2 =_ 84.0 (79.3-92.6) prescribed to the median isodose of 72% (60-80%) distribution. Additionally, the median maximum dose of the PTV was 77.8 (43.3-91.8) Gy. The dose information for other related organs (bronchial tree, trachea, spinal cord, heart, esophageal, and Load lung) is provided in **Table 2**.

**Table 2:** Treatment characteristics of the 58 patients.

Treatment characteristics	Median (range) N = 58
Max dose (Gy)	
PTV	77.8 (43.3,91.8)
Bronchial tree	56.4 (7.0,69.8)
Trachea	25.0 (1.5,61.8)
Spinal cord	16.6 (1.3,43.3)
Heart	47.9 (.44,64.5)
Esophageal	31.06 (3.7,53.7)
Load lung	67.4 (14.4,7.7)
Fractions, n (%)	
6 fractions	2 (3.5%)
7 fractions	31 (53.4%)
8 fractions	25 (43.1%)
BED_10_	100.8 (95.2,111.5)
EQD_2_	84.0 (79.3,92.6)
Isodose line (%)	72 (60,80)

PTV, planning target volume; BED_10_, biological equralent dose; EQD_2,_ equivalent Dose in 2 Gy/f.

### OS, PFS, and LC

Univariate analyses showed that patients with PTV<53.0 cm³ experienced significantly longer OS times (P=0.020), as shown in **Table 3**. In multivariate analyses, PTV and maximum dose of Load lung were significant prognostic factors for OS (all P <0.05, **Table 4**).

**Table 3:** Univariate analysis of factors associated with death.

	χ2	P-value
Sex (male vs female)	0.064	0.800
Age (≥75 vs <75 years)	0.351	0.554
KPS (≥80 vs<80)	1.742	0.783
ECOG (0-2 vs ≥3)	2.313	0.128
Smoke (yes vs no)	1.362	0.243
Pre-SBRT symptoms		
None	1.574	0.210
Cough	0.386	0.534
Hemoptysis	0.411	0.521
Histology (squamous vs adenocarcinoma vs others)	0.047	0.977
T stage (T1 vs T2a vs T2b vs T3)	0.910	0.634
PTV (cm³) (≥53.0 vs <53.0)	5.523	**0.020**
Before therapy (no vs yes)	0.641	0.423
Max dose (Gy)		
PTV (<85.93 vs ≥85.93)	3.133	0.077
Bronchial tree (≤59.0 vs >59.0)	0.183	0.669
Esophageal (≤23.5 vs >23.5)	0.620	0.431
Trachea (≤2.2 vs >2.2)	1.754	0.185
Load lung (≥64.2 vs <64.2)	0.503	0.478
Fractions (6 vs 7 vs 8)	0.514	0.474
BED_10_ (Gy), (≥98 vs <98)	0.514	0.474
Isodose line% (≥65.5 vs <65.5)	0.747	0.388

PTV, planning target volume; BED10, biological equralent dose; EQD2; Equivalent Dose in 2 Gy/f.Bolded figures highlight p-values lower than 0.05, indicating significant differences between the the indicated tumor cohorts.

**Table 4:** Multivariate analyses of predictors of OS in patients with UCLC treated with SBRT.

Variables	P-value	Hazard ratio (95% CI)	95% Confidence interval	
			Lower	Upper
Sex (male vs female)	0.442	1.808	0.399	8.190
Age (≥75 vs <75 years)	0.631	1.509	0.282	8.080
KPS (≥80 vs <80)	0.709	1.016	0.936	1.103
ECOG (0-2 vs ≥3)	0.541	1.578	0.366	6.812
Smoke (yes vs no)	0.217	0.328	0.056	1.923
Pre-SBRT symptoms (yes vs no)	0.121	0.217	0.032	1.496
T stage (T1 vs T2 vs T3)	0.121	0.380	0.112	1.293
PTV (cm³) (≥53.0 vs <53.0)	**0.004**	42.142	3.328	533.634
Max dose (Gy)				
PTV (<85.93 vs ≥85.93)	0.292	3.676	0.327	41.325
Bronchial tree (≥59.0 vs <59.0)	0.306	2.534	0.427	15.053
Oesophageal (≥23.5 vs <23.5)	0.874	1.155	0.194	6.867
Trachea (≥2.2 vs <2.2)	0.186	2.630	0.627	11.027
Load lung(≥64.2 vs <64.2)	**0.012**	0.170	0.042	0.680
BED_10_ (≥98 vs <98)	0.238	0.307	0.043	2.184
Isodose line% (≥65.5 vs <65.5)	0.647	0.494	0.024	10.090

PTV, planning target volume; BED_10_, biological equivalent dose. Bolded figures highlight p-values lower than 0.05, indicating significant differences between the the indicated tumor cohorts.

**Figure 1:**
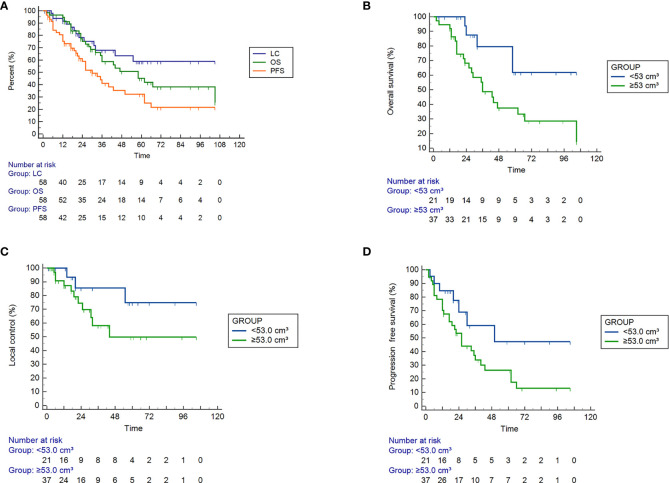
Efficacy of SBRT in 58 patients with ultra-central lung cancer determined by Kaplan ‐ Meier curves. **(A)** Overall survival (OS), tumor local control (LC), and progression ‐ free survival (PFS) rates of all patients, P = 0.007. **(B–D)** The OS (P = 0.019), LC (P = 0.138) and PFS (P = 0.058) rates for tumor volumes <53.0 cm³ and > = 53.0 cm³.

As shown in **Figures 1A, B**, the 1-year, 2-year and 5-year OS rates were 94.7%, 75.0%, and 45.0% for all patients; 100%, 87.5%, and 61.6% for PTV<53.0 cm³; and 91.9%, 68.1%, and 37.4% for PTV ≥53.0 cm³, respectively (P<0.05). As shown in **Figures 1A, C**, the 1-year, 2-year and 5-year LC rates were 91.5%, 78.0%, and 58.6% for all patients; 100%, 85.2%, and 74.6% for PTV <53.0 cm³; and 86.9%, 74.2%, and 49.6% for PTV ≥53.0 cm³, respectively. As shown in **Figures 1A, D**, the 1-year, 2-year, and 5-year PFS rates were 75.2%, 58.7% and 32.3% for the entire cohort; 84.6%, 68.6%, and 47.1% for PTV <53.0 cm³; and 70.3%, 53.4%, and 26.3% for PTV ≥53.0 cm³, respectively. The OS, LC and PFS of patients with PTV <53.0 cm³ were obviously better than those of patients with PTV ≥53.0 cm³.

### Toxicities

After receiving SBRT, only 2 (3.5%) patients experienced grade ≥3 cancer-related toxicities. One patient with dyspnea had grade 5 radiation pneumonitis and died 2 years after radiotherapy (**Figure S1A**). Although one patient died of sudden hemoptysis 31 months after radiotherapy, there was no strong evidence to clarify that the event was related to SBRT treatment (**Figure S1B**). No esophagitis was observed in any of the patients. All the toxic events and adverse events (grade ≥3) after SBRT are shown in **Supplementary Tables S1**, **S2** respectively.

## Discussion

Compared with other treatments, SBRT has greater dose conformality to the tumor contour and a sharp dose gradient to realize a more precise treatment (14, 15). Although UCLC is commonly treated with SBRT, there are still some controversies regarding its efficacy and toxicity. Therefore, we retrospectively examined 58 patients with UCLC in our single-central institution. Except for two patients who received a dose of 56 Gy/6f, the other patients received a dose of 56 Gy in 7 or 8 fractions. From this study, we observed that patients with PTV <53.0 cm³ showed better OS than those with a large PTV. At a median follow-up of 57 months 1-, 2- and 5-year OS rates were 94.7%, 75.0%, and 45.0%, respectively. Local control rates at 1, 2 and 5 years were 91.5%, 78.0%, and 58.6%, respectively. 3.5% of patients experienced grade ≥3 cancer-related toxicities. Even though the survival of patients with adenocarcinoma has been indicated to be better (16), there were no significant differences in OS, LC, or PFS for histological types in our study. This may have resulted from the unavailability of histological results in 27 (46.6%) cases.

Compared with the results of Yoshiko Oshiro1 et al. (17),our results, especially the results of PTV <53 cm³ (2-year OS and PFS rates of 87.5% and 68.6%, respectively), were significantly better than their results. The outcomes of Yoshiko Oshiro1 et al. (2-year OS and PFS rates of 62.2% and 59.6%, respectively) may be attributed to the fact that 20 of the 21 patients had stage IV tumors or recurrent tumors. In 2015, Chaudhuri et al. (18) published a retrospective study that included 68 patients with lung cancer after SBRT treatment at a dose of 56 Gy in 4/5 fractions. In terms of the 2-year OS (80%), local control (100%), and even radiotherapy-related toxicities (0%), there were no distinct differences between their UCLC group (adjacent to the central airways group) and other groups (peripheral and central group). In 2016, Tekatli et al. (19) reported a study focused on ultra-central lung tumors. They defined UCLC as PTV overlapping with important organs, including the trachea or main bronchus. The median OS and 3-year survival were 15.9 months and 20.1%, respectively, and no local recurrences were found. It is worth noting that 15% of patients developed fatal pulmonary hemorrhage after treatment with 60 Gy in 12 fractions, and grade ≥3 toxicities were observed in 38% of the patients. All the above toxicity outcomes were extremely inconsistent with our results and those of previous reports. The volume of tumors in this study, which was commonly over 5 cm, was relatively larger than ours. In 2018, Srinivas Raman et al. (20) retrospectively reported a study of 206 patients, who were divided into two groups: central and ultra-central lung tumors after SBRT of 60 Gy in 8 fractions (53.9%) or 48 Gy in 4 fractions (29.1%). Finally, their study showed no significant differences in terms of survival, recurrence rates, and toxicities (grade ≥2) between the two groups, and no grade ≥4 toxicities occurred. Chang JH et al. (21) also studied a total of 107 patients with lung cancer: primary or metastatic lung central tumors (61 cases) and ultra-central tumors (46 cases) after five-fraction irradiation. The 2-year OS rates were 57.7% and 50.4% for central and ultra-central tumors, respectively. The two-year local failure and 2-year grade ≥ 3 toxicity rates between the two groups were not similarly identified. In 2019, H‐H Wang et al. (22) studied 37 inoperable T1‐2N0M0 ultra‐central NSCLC patients. Their results stress that the smaller PTV in their cohort may contribute to this finding and confirmed our experimental results in their reports. Together, all the above public studies indicate that further investigation should focus on the radiation scheme to improve tumor control. Simultaneously, the possibility of evaluating in the future the adoption of treatments with partial variations of the prescription dose in volumes overlap with critical structures, as described in the literature in a prospective study that evaluates a simultaneous integrated protection approach (SIP) (23).

All the above reports referring, the UCLC group and central group had similar OS and LC in their own series. One distinction was the toxicity rates of UCLC patients: The grade ≥3 toxicities occurring of two patients in our series: one patient underwent radiation pneumonitis and the other one had hemoptysis. Pneumonitis after SBRT is the most common toxicity. In present study (19) showed that grade ≥ 3 toxicities accounted for 38%, and their study showed obvious grade 5 toxicities. From this, we could learn that patients with tumors with a diameter of more than 5 cm are more likely to experience therapeutic toxicity. For fatal hemoptysis, central tumor location is a critical factor. Another research concluded that a mean dosage to the major bronchus of 91 Gy significantly increased the probability of grade ≥3 toxicity (24). Recently, the HILUS-Trial suggested that the dose to the combined structure’s main bronchi and trachea, as well as the distance of the tumor from the main bronchi, were significant risk factors. Their dosage modeling also revealed that the minimum dose to the “hottest” structural major bronchi and trachea of 0.2 cc was the strongest indicator of lethal bronchopulmonary hemorrhage (25). These findings suggest that we should pay more attention to the occurrence of complications and look for corresponding predictors for radiation toxicity.

## Strengths and Limitations

The limitations of our study are as follows. First and commonly, this is a retrospective study that has inherent limitations of date collections and case retrospection. Additionally, some dead patients might have had treatment-related toxicities. However, they were considered dying from cancer progression or comorbidities. Furthermore, before developing radiotherapy-related toxicities, some patients with distant metastases died of cancer progression. Last, there are still some additional factors that may be related to the evaluation index, and more work is urgently needed to explicate their correlations with toxicity.

The worthy strengths of our study are as follows. I. This is the largest single-gross dose study in a single institution, which avoids the influence of various doses on outcomes. II. PTV, as a potential factor, determines the outcomes of patients and may play a role in guiding the adjustment of the tumor treatment scheme. III. We used relatively high isodose lines so that the tumor lesions received higher radiation doses and the surrounding normal tissues were protected.

## Conclusions

Patients with ultra-central lung cancer can benefit from stereotactic body radiotherapy with a dose of 56/6-8f Gy. Smaller PTV was associated with superior outcomes, and the cure difference needs to be validated by prospective comparative trials.

## Data Availability Statement

The original contributions presented in the study are included in the article/**Supplementary Material**. Further inquiries can be directed to the corresponding authors.

## Ethics Statement

The studies involving human participants were reviewed and approved by Tianjin medical university cancer institute and hospital ethics board. The patients/participants provided their written informed consent to participate in this study. Written informed consent was obtained from the individual(s) for the publication of any potentially identifiable images or data included in this article.

## Author Contributions

ZY and ZT conceived and designed the study. BW carried out the experiments, analyzed the date and wrote the manuscript. YD, XY, FL, and YS designed treatment plans. JW, HC, and ZN participated in the execution of treatment. All authors read and approved the final version of the manuscript.

## Funding

This work was supported by grants from The National Natural Sciences Foundation of China (Grant nos. 81602678 and 82073356) and The Science & Technology Development Fund of Tianjin Education Commission for Higher Education, Grant no. 2021ZD034.

## Conflict of Interest

The authors declare that the research was conducted in the absence of any commercial or financial relationships that could be construed as a potential conflict of interest.

## Publisher’s Note

All claims expressed in this article are solely those of the authors and do not necessarily represent those of their affiliated organizations, or those of the publisher, the editors and the reviewers. Any product that may be evaluated in this article, or claim that may be made by its manufacturer, is not guaranteed or endorsed by the publisher.

## Supplementary Material

Supplementary Figure 1 Radiation plan images of patients with grade ≥3 toxicities. **(A)** Patient 1 with dyspnoea had radiation pneumonitis and died 24 years after radiotherapy. **(B)** Patient 2 died of sudden massive hemoptysis 31 months after radiotherapy.
