# Supplementary material for: Safety and Efficacy of Stereotactic Ablative Radiotherapy for Ultra-Central Lung Cancer
Source: Front Oncol. 2022 Apr 29;12:868844. doi: 10.3389/fonc.2022.868844 (PMC9118536; doi:10.3389/fonc.2022.868844)
Supplement: Supplementary Figure 1 — Radiation plan images of patients with grade ≥3 toxicities. (A) Patient 1 with dyspnoea had radiation pneumonitis and died 24 years after radiotherapy. (B) Patient 2 died of sudden massive hemoptysis 31 months after radiotherapy. [file Table_1.docx]

Supplementary Table S1. Related acute and late adverse events after SBRT

| Adverse events | Acute | | Late | |
| --- | --- | --- | --- | --- |
| Characteristic | Grade <3 | Grade ≥3 | Grade <3 | Grade ≥3 |
| Pneumonitis | 15 (26%) | 0 | 1 (1.7%) | 1 (1.7%) |
| Hemoptysis | 0 | 0 | 0 | 1 (1.7%) |
| Fatigue | 0 | 0 | 0 | 0 |
| Pulmonary fibrosis | 1 (1.7%) | 0 | 1 (1.7%) | 0 |
| Bronchial stenosis | 3 (5.2%) | 0 | 1 (1.7%) | 0 |
| Bronchial occlusion | 2 (3.5%) | 0 | 1 (1.7%) | 0 |
| Pleural effusion | 0 | 0 | 1 (1.7%) | 0 |
| Hoarseness | 0 | 0 | 1 (1.7%) | 0 |

Supplementary Table S2. Characteristics of patients experiencing grade ≥3 toxicities

| Patient | Patient 1 | Patient 2 |
| --- | --- | --- |
| Sex | Male | Female |
| Age | 77 | 73 |
| Smoke | Yes | No |
| History | SSC | unknown |
| T stage | T2a | T1a |
| Pre-SBRT symptoms | G1 cough | G1 cough and breathless |
| PTV (cm^3^) | 75.7 | 42.7 |
| PTV Dmax (Gy) | 77.8 | 77.8 |
| BED10 | 95.2 | 95.2 |
| Isodose line% | 0.72 | 0.72 |
| Toxicity | G5 RP | G5 Hemoptysis |
| Status | Death | Death |
| Cause of death | RP | Sudden hemoptysis |
| Location | LUL | RH |

SSC: small cell lung cancer; RP: radiation pneumonia; RUL: right upper lobe;

RH: right hilar LUL: left upper lobe.
